# Bexarotene-induced cell death in ovarian cancer cells through Caspase-4-gasdermin E mediated pyroptosis

**DOI:** 10.1038/s41598-022-15348-7

**Published:** 2022-07-01

**Authors:** Tatsuya Kobayashi, Akira Mitsuhashi, Piao Hongying, Masashi Shioya, Katsushi Kojima, Kyoko Nishikimi, Kinnosuke Yahiro, Makio Shozu

**Affiliations:** 1 Department of Reproductive Medicine, Graduate School of Medicine, Chiba University, Inohana 1-8-1, Chuo-ku, Chiba, 260-8670 Japan; 2 Department of Obstetrics and Gynecology, School of Medicine, Dokkyo Medical University, Tochigi, 321-0293 Japan; 3 Takahashi Women’s Clinic, Chiba, 260-0028 Japan; 4 Department of Microbiology and Infection Control Sciences, Division of Biological Sciences, Kyoto Pharmaceutical University, Kyoto, 607-8412 Japan

**Keywords:** Chemotherapy, Ovarian cancer

## Abstract

Bexarotene selectively activates retinoid X receptor, which is a commonly used anticancer agent for cutaneous T-cell lymphoma. In this study, we aimed to investigate the anticancer effect of bexarotene and its underlying mechanism in ovarian cancer in vitro. The ES2 and NIH:OVACAR3 ovarian cancer cell lines were treated with 0, 5, 10, or 20 µM of bexarotene. After 24 h, cell number measurement and lactate dehydrogenase (LDH) cytotoxicity assay were performed. The effect of bexarotene on CDKN1A expression, cell cycle-related protein, cell cycle, pyroptosis, and apoptosis was evaluated. Bexarotene reduced cell proliferation in all concentrations in both the cells. At concentrations of > 10 µM, extracellular LDH activity increased with cell rupture. Treatment using 10 µM of bexarotene increased *CDKN1A* mRNA levels, decreased cell cycle-related protein expression, and increased the sub-G1 cell population in both cells. In ES2 cells, caspase-4 and GSDME were activated, whereas caspase-3 was not, indicating that bexarotene-induced cell death might be pyroptosis. A clinical setting concentration of bexarotene induced cell death through caspase-4–mediated pyroptosis in ovarian cancer cell lines. Thus, bexarotene may serve as a novel therapeutic agent for ovarian cancer.

## Introduction

Ovarian cancer is the fifth most common cause of cancer-related death and the most deadly gynecological cancer in western countries^1^. Although the initial clinical response is generally satisfactory, more than 70% of affected patients experience recurrences and ultimately die. Multimodality treatment with cytoreductive surgery and platinum–taxane-based chemotherapy combined with molecularly targeted drugs has shown prolonged survival. However, the overall cure rate of the disease has not considerably changed. Therefore, additional treatment strategies that can improve survival are urgently required.

Retinoid X receptor (RXR) is a member of nuclear receptor family. RXR acts as a transcriptional factor by homodimerizing with itself or other nuclear receptor families when stimulated by their specific ligands. Bexarotene (LGD1069, Targretin®) is an RXR-selective agonist that binds and activates all three RXR isoforms (RXRα, RXRβ, and RXRγ) with equivalent affinity and potency^2^. Bexarotene is approved by the FDA and is a widely used drug for treating cutaneous manifestations in cutaneous T-cell lymphoma (CTCL)^3^. Moreover, this drug has gained increasing attention in cancer treatment; hence, it has been used as an off-label drug for non-small cell lung cancer^4^ and breast cancer^5^. In vitro studies have shown that bexarotene can also prevent and overcome acquired drug resistance in advanced breast cancer^6,7^, non-small cell lung cancer^8,9^, and even advanced prostate cancer^10^. An in vitro analysis revealed that bexarotene could kill cancer cells via apoptosis, decrease TGF-α and EGFR expressions, induce cellular senescence associated with increased p21 and p16 expressions, and promote G1 phase cell cycle arrest^11^. However, the precise mechanism of cell proliferation inhibition by bexarotene remains unclear.

Pyroptosis is one of the regulated cell deaths triggered by certain caspases that are mainly activated by inflammation, anticancer drugs, and endoplasmic reticulum (ER) stress. Inflammatory caspases (caspase-1/4/5 in humans) cleave gasdermin D (GSDMD), which is required and sufficient for pyroptosis^12,13^. The polymerized GSDMD N-terminal forms pores in the cell membrane to increase permeability and osmosis, thereby causing cell rupture, which is a characteristic of pyroptosis. Caspase-3, a well-known apoptotic caspase, causes pyroptosis with gasdermin E (GSDME/DFNA5). Chemotherapy drugs such as cisplatin, etoposide, and doxorubicin induce pyroptosis in tumor cells with high GSDME levels caused by caspase-3^14^. Several anticancer drugs, such as chemotherapy drugs and miRNA, can reduce tumor viability and invasiveness by inducing tumor pyroptosis^15^. Hence, pyroptosis induction might be a potent anticancer drug target^16^.

In epithelial ovarian cancer, retinoic acid receptors are frequently and strongly expressed and may indicate an adverse prognosis^17^. Therefore, RXR-selective retinoids, such as bexarotene, might potentially treat ovarian cancer; however, the effect of bexarotene on ovarian cancer remains unclear. Hence, this study aimed to investigate whether bexarotene could suppress the proliferation of ovarian cancer cells in vitro and better understand the antitumorigenic potential of bexarotene by exploring the precise mechanism of bexarotene on cell death, particularly pyroptosis.

## Methods

### Cell culture

The ovarian clear cell carcinoma cell line (ES2) and serous ovarian carcinoma cell line (NIH:OVACAR3) were used. ES2 and NIH:OVACAR-3 cell lines were purchased from ATCC (Manassas, VA, USA) and RIKEN BioResource Center (Tsukuba, Japan). These ovarian cancer cell lines were routinely cultured in RPMI 1640 (Nacalai Tesque, Kyoto, Japan) containing inactivated 2% fetal bovine serum (Equitech-Bio, Kerrville, TX, USA) and 1% penicillin–streptomycin (Nacalai Tesque) at 37 °C with 5% CO_2_ in room air.

### Cell proliferation assay and lactate dehydrogenase (LDH) cytotoxicity assay

In the culture medium, we seeded the cells in 12-well plates (100,000 cells/well). At 80% confluence, the cells were treated with 0–20 µM of bexarotene (Tokyo Chemical Industry Co., Ltd., Tokyo, Japan) dissolved in dimethyl sulfoxide (Fujifilm Wako, Osaka, Japan) for 24 h. Bexarotene is insoluble in water; hence, it was dissolved in dimethyl sulfoxide (DMSO). Therefore, all the experiments, including the untreated control, were performed with a concentration of 0.1% DMSO. The cells were then treated with trypsin–EDTA solution (Nacalai Tesque) and mixed with the total amount of trypan blue solution (Nacalai Tesque). Subsequently, the live cells were counted using a cell-counting chamber (WakenBtech Co., Ltd., Kyoto, Japan) under a stereomicroscope (Olympus, Tokyo, Japan).

Moreover, we collected the cell culture supernatant and measured the LDH activity using the LDH Cytotoxicity Assay Kit (Nacalai Tesque) according to the manufacturer’s protocol. The LDH levels of the cell culture supernatant derived from the bexarotene-treated cell group were normalized relative to the cultured medium derived from the control group (vehicle of the bexarotene-treated group). Each experiment was repeated at least three times, and the LDH activity was compared to that of the control group.

### Protein extraction and western blot

We washed the cultured cells with PBS and extracted the total protein using a complete Lysis-M reagent (Roche, Basel, Switzerland) containing 1% Halt™ Phosphatase Inhibitor Cocktail (Thermo Fisher Science, Waltham, MA, USA) for 15 min at room temperature. We subsequently centrifuged the cell lysate at 15,000×*g* for 15 min at 4 °C, separated 7.5 µg of total protein in 10% or 4–15% gradient SDS-PAGE (Bio-Rad, Hercules, CA, USA), and transferred it to a polyvinylidene fluoride (PVDF) membrane (Merck Millipore, Burlington, MA, USA). Nonspecific binding to the PVDF membrane was blocked in Blocking one-P solution (Nacalai Tesque) at room temperature for 30 min. The first antibody was reacted at 4 °C for overnight. Subsequently, we washed the PVDF membrane in PBS-T and enhanced the protein signal by anti-mouse or rabbit IgG antibody (1/10,000 or 5000) at room temperature for 1 h. We used ECL select (Roche) or ECL Prime reagent (Roche) to detect protein signals. A densitometric analysis of western blot was performed using a densitometer (CS analyzer version 3.0 software, ATTO, Tokyo, Japan), and the intensity was normalized to the b-actin protein levels.

### RNA extraction and real-time quantitative PCR

Total RNA from cultured cells was extracted using the RNeasy Mini Kits (Qiagen, Hilden, Germany) according to the manufacturer’s protocol. Using the SuperScript VILO cDNA Synthesis Kit (Thermo Fisher Science, Waltham, MA, USA), we reverse-transcribed the extracted RNA into complementary DNA (cDNA). Real-time quantitative PCR (RT-qPCR) was conducted using Light Cycler DNA Master SYBR Green I Kit (F. Hofmann–Roche Ltd., Basel, Switzerland) with Light Cycler Nano System (F. Hofmann–Roche Ltd). Furthermore, we evaluated the *CDKN1A* and *GAPDH* (internal control) mRNA expression levels. The expression levels of measured genes were normalized relative to that of *GAPDH* mRNA*.* The 2^−∆∆Cq^ method was used to obtain the relative quantitative value^18^. Supplementary Table S1 lists the gene-specific primers.

### Cell cycle analysis

Vehicle or 10 µM–bexarotene-treated cells were cultured for 48 h and then harvested by trypsin–EDTA solution. The cells were washed twice with PBS and fixed with 70% ethanol (Fujifilm Wako) at –30 °C until cell cycle analysis. Following this, cells were incubated in 20 µg/ml RNase for 30 min after staining with 50 µg/ml propidium iodide (PI) solution (Nacharai tesque) at 4 °C for 30 min in darkness. Fluorescence intensity was analyzed using CytoFLEX flow cytometer (BECKMAN COULTER, Brea, CA, USA) with CytEXpert Soft wear (BECKMAN COULTER). We analyzed 15,000 events to determine the cell cycle phase and classified cells into sub-G1, G0/G1, S, or G2/M phases according to the fluorescence intensity of the cells.

### Small interfering RNA (siRNA) transfection

Using Lipofectamine RNAiMAX (Thermo Fisher Science, Waltham, MA, USA), we transfected ES2 cell lines with a siRNA. The si-protein kinase R-like ER kinase (PERK; SI02223718; Qiagen, Hilden, Germany) RNA, si-inositol-requiring enzyme 1-A (IRE1A; SI00605255; Qiagen), or a non-target siRNA (control) were used. To prepare the siRNA transfection solution for each tube, we mixed 5 pmol of siRNA with 50 μL of Opti-MEM reduced-serum medium (Thermo Fisher Science, Waltham, MA, USA). Concurrently, we mixed 1 μL of Lipofectamine RNAiMAX with 50 μL of Opti-MEM. Following this, the two solutions were mixed by gentle pipetting and incubated for 5 min at room temperature to allow siRNA/lipid complexes to form. The treated cells forming siRNA/lipid complexes were then incubated for 48 h at 37 °C, followed by bexarotene treatment.

### Antibodies and other reagents

For detecting the target protein, we used an anti-caspase-4 antibody (1/1000; MBL, Tokyo, Japan), anti-caspase-3 (D175) antibody (1/1000; Cell Signaling Technology, Danvers, MA, USA), anti-GSDME antibody (1/1000; Proteintech Group, Rosemont, IL, USA), anti-cyclin D1 antibody (1/1000; Cell Signaling Technology), anti-Rb antibody (1/1000; Cell Signaling Technology), anti-phospho-Rb antibody, anti-CDK4 antibody (1/1000; Cell Signaling Technology), anti-CDK6 antibody (1/1000; Cell Signaling Technology), anti-PERK antibody (1/1000; Cell Signaling Technology), and anti-β actin antibody (1/5000; Cell Signaling Technology) as the first antibodies.

We used ZYVAD-FMK (R&D SYSTEMS, Minneapolis, MN, USA) dissolved in dimethyl sulfoxide (Sigma-Aldrich, St. Louis, MO, USA) for caspase-4 inhibition experiments.

### Statistical analysis

The cell proliferation assay was statistically analyzed using an independent *t*-test. All comparisons were performed using a two-sided test. In addition, *P* < 0.05 was considered statistically significant. All statistical data were analyzed using the JMP statistical software (SAS Institute Inc., Cary, NC, USA).

## Results

### Bexarotene reduced cell viability and affected cell shape

**Figure 1:**
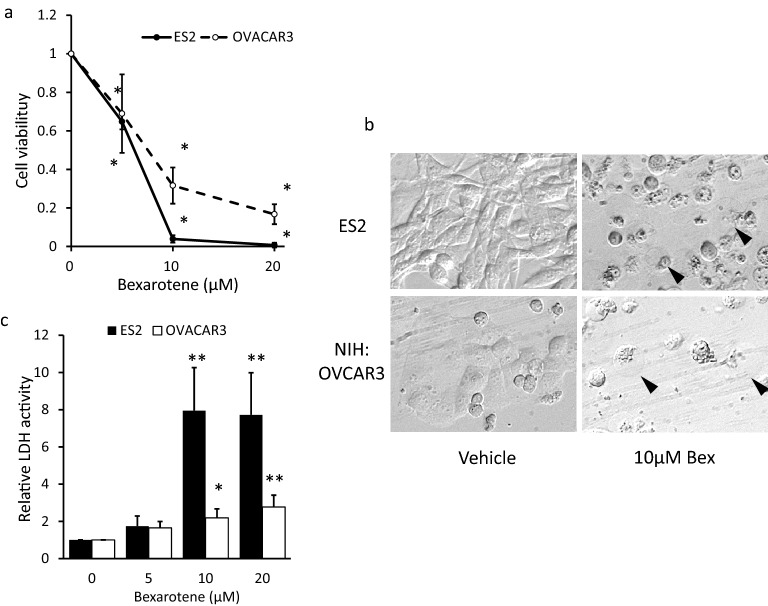
Bexarotene treatment reduced cell viability and induced cell death by LDH release and plasma membrane bursting. (**a**) In ES2 and NIH:OVACAR3 cells, counted manually were cell numbers after 24 h of bexarotene treatment. Measured was the cell viability against the bexarotene nontreated group. The results presented were the mean, ± standard deviation for at least three independent experiments. (**b**) Morphological analysis of ES2 cells (upper two panels) and NIH:OVACAR3 (lower two panels). The plasma membrane expanded, and the broken cells had a swollen structure (arrow). (**c**) bexarotene treatment was extracellular LDH activity. The results presented were the mean ± standard deviation for at least three independent experiments. *p < 0.05, **p < 0.01, compared with bexarotene-free control. LDH, lactate dehydrogenase.

First, we examined the effect of bexarotene treatment on cell viability, cell membrane damage, and cell morphological shape to determine whether bexarotene had anticancer potential against ovarian cancer cell lines. After 24 h of bexarotene treatment, cell viability was significantly reduced dose-dependently in both the ES2 and NIH:OVACAR3 cells (Fig. 1a), and the morphology in cancer cells drastically changed, as observed microscopically (Fig. 1b). Furthermore, bexarotene-treated cells were detached from the surface of the culture dish, and the plasma membrane clearly expanded. Moreover, some cells exhibited a post-cell burst appearance (≥ 10 µM of bexarotene).

The effect of bexarotene on plasma membrane damage was evaluated by measuring the released LDH activity in the culture supernatant. In living cells, LDH is present in the cytoplasm, but it is released extracellularly due to cell membrane changes that occur during the cell death processes, such as in necroptosis and pyroptosis^19^. The levels of released LDH significantly increased in the 10 and 20 µM–bexarotene-treated group compared with the control group in ES2 cells (Fig. 1c, *P* < 0.05). Likewise, the LDH levels in NIH:OVACAR3 cells also significantly increased in the 10 and 20 µM–bexarotene-treated group (*P* < 0.05). These results indicated that bexarotene induced cell death by causing plasma membrane damage in ovarian cancer cell lines.

### Bexarotene increased p21 mRNA expression and induced cell growth inhibition with sub-G1 arrest

Tanaka et al. reported that a ligand-activated RXR homodimer upregulates cyclin-dependent kinase inhibitor p21 (CDKN1A) expression by directly binding to its promoter region and regulating cell proliferation^20^. Therefore, we evaluated whether bexarotene reduced the cell viability by acting as an RXR agonist in ovarian cancer cell lines.

**Figure 2:**
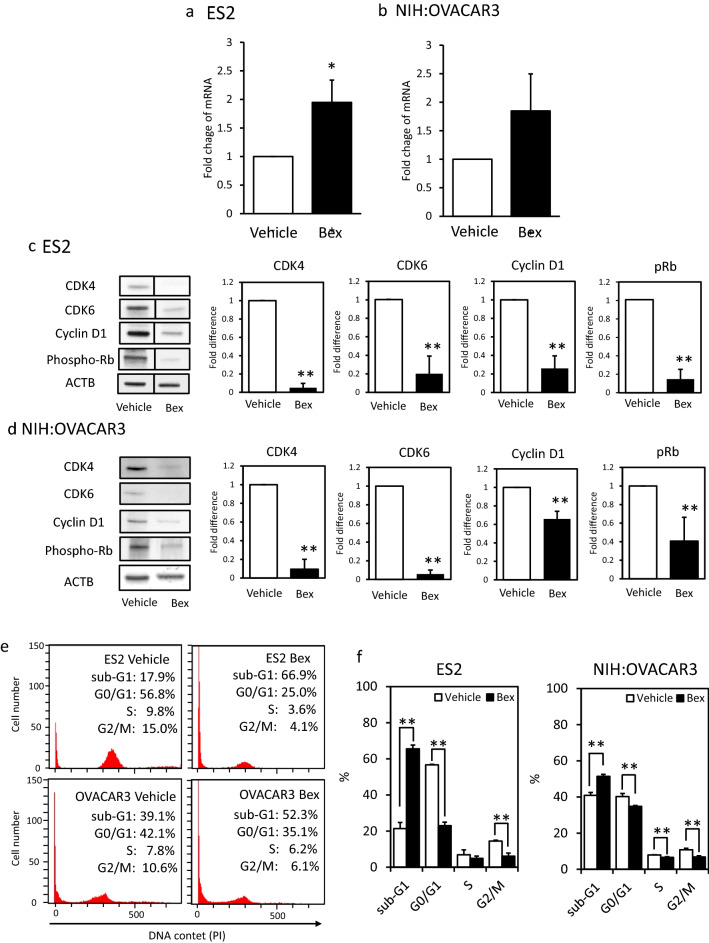
Bexarotene treatment increased CDKN1A mRNA expression, decreased cell cycle-related proteins, and increased the sub-G1 phase cell population. Levels of CDKN1A mRNA were measured and compared between 10 µM bexarotene-treated cells and nontreated cells 24 h after bexarotene treatment in (**a**) ES2 and (**b**) NIH:OVACAR3 cells. CDKN1A expression was assessed using the 2-ΔΔCT quantitation method and normalized to housekeeping gene GAPDH using a no-treatment control as a calibrator. The results presented were mean, ± standard deviation for at least three independent experiments. (**c**, **d**) Western blot analysis was performed to evaluate the effect of 10 µM bexarotene on cell cycle-related proteins (CDK4, CDK6, cyclin D1, and phospho-Rb) in ES2 and NIH:OVACAR3 cells. Used as an internal control was β-actin. Columns and bar graphs represent the mean ± standard deviation for at least three independent experiments. Quantification of protein levels was performed by densitometry and normalized to β-actin. (**e**, **f**) Cell cycle analysis with PI-stained cells. Bexarotene (10 µM ) treatment significantly increased the sub-G1 phase cell population and reduced G0/G1 and G2/M phase population in both the cell lines. White bars and the black bars represent the data in the vehicle- and 10 μM–bexarotene treated cell, respectivery. *P < 0.05, **P < 0.01, compared with bexarotene-free control. ACTB, β-actin.

First, we measured the mRNA expression levels of *RXR-α*, *RXR-β*, and *RXR-γ* in ES2 and NIH:OVACAR-3 cells using RT-PCR to confirm the RXR expression. The *RXR-α* and *RXR-β* mRNA were expressed, but *RXR-γ* mRNA was not in both cells (data not shown). Then, to determine whether bexarotene acted as an RXR agonist in ovarian cancer cell lines, we measured the *CDKN1A* mRNA expression levels after 24 h of bexarotene treatment. In both the cell lines, the administration of 10 µM bexarotene increased *CDKN1A* mRNA expression by approximately twofold (Fig. 2a, b).

Subequently, the levels of cell cycle-related proteins, specifically the downstream of p21 protein, CDK4, CDK6, Cyclin D1, and phospho-RB, were examined using western blot in 10 µM–bexarotene-treated cells^21^. After 48 h of bexarotene treatment, the levels of CDK4, CDK6, Cyclin D1, and phospho-RB decreased in both ovarian cancer cell lines (Fig. 2c, d, S2).

Finally, we performed cell cycle analysis with both the PI-stained cell lines after 48 h from bexarotene treatment using flow cytometry analysis. Bexarotene treatment significantly increased the population of the sub-G1 phase and reduced the population of G0/G1 and G2/M phase cells in both the cell lines (Fig. 2e, f).

### Bexarotene-induced pyroptosis via caspase-4 and GSDME-related pathway

**Figure 3:**
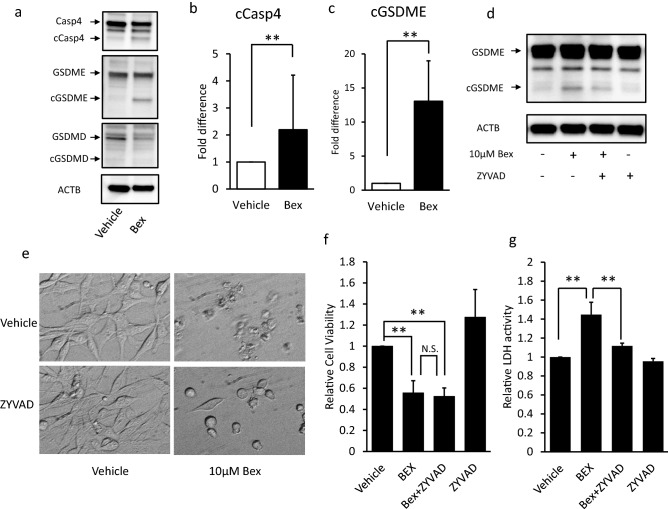
Bexarotene induces pyroptosis in ES2 cells. (**a**) Bexarotene increased pyroptosis-related proteins (Casp4, GSDME, and GSDMD) following exposure with 10 µM of bexarotene for 24 h in ES2 cells. Bexarotene increased cleaved caspase-4 levels (**b**) and cleaved GSDME levels (**c**). Β-actin was used as an internal control. (**d**) ZYVAD-FMK treatment reduced the levels of cleaved GSDME 4 h following bexarotene treatment. ES2 cells were pretreated with or without 10 µM–ZYVAD-FMK (caspase-4 inhibitor) 1 h before 10-µM-bexarotene treatment. (**e**) Bexarotene-treated cells showed pyroptotic-like morphological changes (upper right panel), whereas pretreatment with ZYVAD-FMK attenuated bexarotene-induced cell shape alteration (lower right panel). (**f**) Cell numbers were manually counted 24 h after bexarotene treatment, and cell viability against the bexarotene nontreated group was calculated. Pretreatment with ZYVAD-FMK could not suppress bexarotene-induced cell viability reduction. (**g**) The extracellular LDH levels increased by bexarotene were reduced by pretreatment with ZYVAD-FMK. Columns and bar graphs represent the mean ± standard deviation for at least three independent experiments. **P < 0.01. Casp, Caspase; ACTB, β-actin; cGSDME, cleaved GSDME; cGSDMD, cleaved GSDMD; ZYVAD-FMK, ZYVAD; LDH, lactate dehydrogenase; N.S., not significant.

As above, bexarotene-treated ovarian cancer cell lines exhibited drastic morphological changes with the release of intracellular LDH. The swelling structure formation and membrane rupture during cell death are related to pyroptosis^12,22^. Thus, the bexarotene-induced cell death might be pyroptosis. We used ES2 cells, which were highly sensitive to bexarotene, to determine whether bexarotene induces pyroptosis in ovarian cancer cells. To determine whether the expression and activation of caspase-1 and -4 are caused by bexarotene stimulation at molecular levels, we performed western blot using an antibody specific to caspase-1 and -4. In ES2 cells, caspase-4 was expressed and activated after 24 h of 10-µM-bexarotene treatment (Fig. 3a, b). Meanwhile, caspase-1 mRNA and protein were not detected in the ES2 cell line (data not shown). Furthermore, Bexarotene stimulation could not activate caspase-3, the apoptosis-related caspase (Fig. S3A). During pyroptosis, the activated caspase cleaves GSDM and the N-terminal domain dimer of GSDM induces cell membrane perforation^23^. Therefore, we also evaluated GSDME and GSDMD cleavage using western blot. From 24 h after the start of bexarotene treatment, we detected the cleaved GSDME (Fig. 3a, c). Meanwhile, bexarotene treatment did not cleave GSDMD (Fig. 3a).

We then evaluated whether 10 µM–ZYVAD-FMK (caspase-4 inhibitor) pretreatment can affect pyroptosis. ZYVAD-FMK reduces the cleaved GSDME levels at 4 h after the bexarotene treatment (Fig. 3d, S2B). These results indicated that cleaved GSDME was related to caspase-4 activation. Subsequently, we evaluated whether 10 µM–ZYVAD-FMK pretreatment can mitigate bexarotene-induced cell viability reduction and LDH release into the culture media after 24 h of bexarotene treatment. The pretreatment of ZYVAD-FMK partially attenuated the bexarotene-induced morphological changes in ES2 cells (Fig. 3e). However, as shown in Fig. 3f, pretreatment with ZYVAD-FMK could not restore bexarotene-induced cell viability reduction (*P* = 0.7742, bexarotene-treated group vs. bexarotene with ZYVAD-FMK–treated group). Meanwhile, pretreatment with ZYVAD-FMK suppressed the increase in extracellular LDH levels induced by bexarotene (Fig. 3g; bexarotene-treated group vs. bexarotene with ZYVAD-FMK–treated group; *P* < 0.05). These results indicate that bexarotene has two functions on ovarian cancer cells: cell proliferation suppression and caspase-4 GSDME–related pyroptosis, and these are independent actions.

### Bexarotene-induced ER stress, which is not associated with pyroptosis

The ER stress might cause pyroptotic cell death in several cells in vitro^24,25^. Thus, we examined whether bexarotene-induced ER stress in ES2 cells. We also evaluated whether bexarotene activated the unfolded protein response (UPR) signaling pathways, which are common ER stress markers, in ES2 cells. In this study, we used PERK phosphorylation, and X-binding protein 1 (XBP1) mRNA splicing as ER stress response markers.

**Figure 4:**
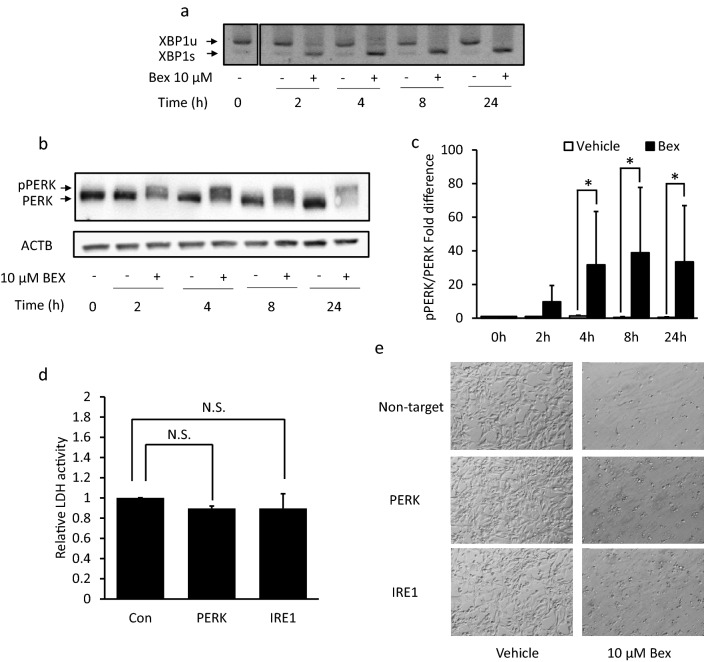
Bexarotene-induced ER stress, which is not associated with pyroptosis. Time-dependent changes in (**a**) XBP1 mRNA and (**b**, **d**) PERK protein levels following exposure to 10 µM of bexarotene for 2–24 h. (**a**) The XBP1 mRNA splicing was upregulated in a time-dependent manner. (**b**, **c**) the molecular weight of PERK was rapidly altered to a higher molecular form (active form). (**d**, **e**) Control, PERK, or IRE1 siRNA-transfected cells were incubated for 24 h with 10 µM of bexarotene. (**d**) After bexarotene treatment, extracellular LDH was measured and compared between the control group and each siRNA-transfer group. No significant differences in LDH activity were found between each siRNA-transfer group and the control group. (**e**) Morphological analysis of control, PERK, or IRE1 siRNA-transfer ES2 cell line. β-actin was used as an internal control. Columns and bar graphs represent the mean ± standard deviation for at least three independent experiments. *P < 0.05, N.S., not significant.

Spliced XBP1 worked as a transcriptional factor for upregulating or downregulating the UPR-related gene under ER stress conditions. From 2 h after bexarotene treatment, *XBP1* mRNA was spliced in an increased time-dependent manner (Fig. 4a, S4A). After 2 h of bexarotene treatment, the molecular weight of PERK was rapidly shifted to a higher molecular form (Fig. 4b, c, S4B), indicating that PERK protein was activated via phosphorylation. These results indicated that bexarotene rapidly induced ER stress in ES2 cells. Subsequently, we examined whether the knockdown of ER stress sensor proteins attenuated the cell death caused by bexarotene in ES2 cells. We found that PERK and IRE1 knockdown could not attenuate the release of LDH (Fig. 4d) and restore cell morphological changes (Fig. 4e) caused by bexarotene treatment.

## Discussion

This study revealed that bexarotene induced cell death in several ovarian cancer cell lines under a clinical setting concentration. Furthermore, this study clarified the following points: (1) bexarotene-induced cell death might be caused via pyroptosis mediated by caspase-4 activation and GSDME cleavage; (2) bexarotene suppresses cell proliferation through sub-G1 cell cycle arrest; and (3) bexarotene rapidly induced ER stress but might not be related to pyroptosis.

Our study confirmed that bexarotene (5–20 μM) induced cell death and inhibited cell proliferation in ovarian cancer cell lines. This experimental concentration of bexarotene almost has the same or lower concentrations than those of the clinical concentration (C_max_: 10.4 μM, Targretin capsules 75 mg, interview form, Minophagen Pharmaceutical Co., Ltd., Zama, Japan). Conversely, the all-trans retinoic acid (ATRA) concentration, which is a well-investigated retinoic acid against ovarian cancer in vitro, has supraphysiologic concentrations. The ATRA concentration (sometimes as high as 10 μM) in in vitro studies was 5–50 times higher than that in the clinical setting (C_max_: 0.18 μM, VESANOID Capsule 10 mg, interview form, Fuji Pharma Co., Tokyo, Japan). ATRA increases apoptosis and decreases cell proliferation, thereby proving effective against several ovarian cancer cell lines^26–29^. In the study by Lokman et al.^29^, the cell survival of two of five serous ovarian cancer cell lines (OVCAR-3 and OV-90) was inhibited by more than 20%; however, proliferation inhibition was weak or almost nonexistent for the other three cells (OAW28, COV318, and COV362). In addition, several ovarian cell lines, such as COV362, COV318, SKOV-3, and CP70, are resistant to ATRA. Contrary to ATRA, bexarotene had the potential for clinical application in ovarian cancer.

We confirmed that bexarotene-induced cell death is pyroptosis mediated by caspase-4 activation and GSDME cleavage. RNA-seq analysis was performed using ES2 cells 24 h after the 10 µM–bexarotene treatment (data not shown). No changes were observed in the caspases, except for caspase-4. Therefore, the other caspases were not involved in the mechanism of pyroptosis induced by bexarotene. As far as we know, this is the first report of bexarotene-induced death of pyroptotic cells in an ovarian cancer cell line. According to a research by Zhang et al., bexarotene induces apoptosis in CTCL cells^30^. Apoptosis is characterized by many distinct morphological features, including cell shrinkage and fragmentation into membrane-bound apoptotic bodies^31^, and mediated by caspase-3 and -8 activation^32^. However, the characteristic morphology of apoptosis was not observed in bexarotene-treated ovarian cancer cell lines in ES2 cells and NIH:OVACAR3 cells. In addition, the extracellular activity of LDH, which is initially present intracellularly, is not elevated on apoptosis. This type of cell death featured cell proliferation, such as the morphological changes during pyroptosis^33^. Pyroptosis is a proinflammatory programmed cell death typically induced by a viral infection, toxin, and chemotherapy drugs^13^. Three main pathways of inducing pyroptosis have been reported. The first is the canonical pathway of pyroptosis, which is regulated by inflammation-activated caspase-1. The second is the non-canonical pyroptotic pathway regulated by caspase-4/5 in humans and caspase-11 in the mouse. GSDMD is a common substrate for caspase-1/4/5/11 for both canonical and non-canonical pyroptosis^23^. The last pathway is mediated by caspase-3 and GSDME, which can be activated by chemotherapy drugs^14^. The study showed that bexarotene induced pyroptotic cell death through the caspase-4–GSDME-dependent signaling pathway in an ovarian cancer cell line. It is the first report demonstrating the induction of pyroptosis by caspase-4 activation and GSDME cleavage.

The anticancer effect of bexarotene was thought to be due to its genomic function as an RXR agonist. The ligand-activated RXR homodimer binds two RXR consensus domains within the promoter region of *CDKN1A* genes and upregulates the *CDKN1A* mRNA expression^20^. In this study, bexarotene upregulated CDKN1A, which codes a cyclin-dependent kinase inhibitor p21, and downregulated cell cycle-related proteins downstream of p21.Therefore, bexarotene also acts as an RXR agonist in ovarian cancer cells. However, several studies indicated that the accumulation of p21 could induce growth inhibition and cell cycle arrest but could not induce cell death^34^. Thus, the induction of CDKN1A alone could not explain bexarotene-induced cell death. However, in this study, bexarotene could induce cell death with characteristic morphological changes of pyroptosis through caspase-4 and GSDME. In addition, the knockdown of RXRα or RXRβ by siRNA could not attenuate the LDH release induced by bexarotene (Supplementary Figure S1). These results suggested that the induction of pyroptosis through caspase-4 and GSDME was not mediated by RXR. Therefore, we speculated that bexarotene has genomic functions via RXR and non-genomic functions via caspase-4 and GSDME. These two actions were also considered independent. Furthermore, the cell-killing effect of bexarotene was observed at a clinical concentration (10 µM); however, CDKN1A activation only showed a twofold change at this concentration. Therefore, bexarotene could have induced cell death by pyroptosis mediated by caspase-4 activation rather than by p21-mediated cell cycle arrest.

In this study, we also showed that the bexarotene-activated ER stress in ovarian cancer cells was consistent with the findings of a previous report wherein ER stress was induced in the human neuroblastoma cell line by a high concentration of bexarotene^35^. Several studies have reported that ER stress can lead to pyroptosis^24,25,36^. Palmitic acid and the ER stressor tunicamycin induce ER stress, leading to pyroptosis. An ER stress inhibitor can inhibit pyroptosis caused by palmitic acid or tunicamycin-induced ER stress. In this study, we have also demonstrated that the attenuation of ER stress by si-PERK and si-ERE1a could not restore bexarotene-induced pyroptosis, suggesting that bexarotene-induced pyroptosis is not mediated by ER stress in ovarian cancer cells. Hence, future research should investigate how bexarotene induces pyroptosis in ovarian cancer cells.

## Conclusions

Our study showed that the clinical concentration of bexarotene reduces cell proliferation by inducing p21, which is still widely known and can also cause cell death, which is possibly pyroptosis, in ovarian cancer cell lines. Therefore, bexarotene is a potential anticancer drug for ovarian cancer. This study, however, was unable to elucidate the mechanisms of bexarotene-induced pyroptosis. Therefore, to better understand the pharmacological action of bexarotene, future research should further investigate the mechanisms of bexarotene-induced pyroptosis in ovarian cancer cells.

## Supplementary Information

Supplementary Information.

## Data Availability

All data analyzed in this study are included in this published article.
