# Supplementary material for: Bexarotene-induced cell death in ovarian cancer cells through Caspase-4-gasdermin E mediated pyroptosis
Source: Sci Rep. 2022 Jul 1;12:11123. doi: 10.1038/s41598-022-15348-7 (PMC9249775; doi:10.1038/s41598-022-15348-7)
Supplement: Supplementary file 1 — Supplementary Information. [file 41598_2022_15348_MOESM1_ESM.pdf]

Bexarotene-induced cell death in ovarian cancer cells through Caspase-4-gasdermin E mediated pyroptosis

Tatsuya Kobayashi[1], Akira Mitsuhashi[1,2]*, Piao Hongying[1], Masashi Shioya[1,3], Katsushi Kojima[3], Kyoko Nishikimi[1], Kinnosuke Yahiro[4], Makio Shozu[1]

[1]Department of Reproductive Medicine, Graduate School of Medicine, Chiba University, Chiba 260-8670, Japan

[2]Department of Obstetrics and Gynecology, School of Medicine, Dokkyo Medical University, Tochigi, 321-0293, Japan

[3]Takahashi Women's Clinic, Chiba 260-0028, Japan

[4]Department of Microbiology and Infection Control Sciences, Division of Biological Sciences, Kyoto Pharmaceutical University, Kyoto 607-8412, Japan

*Corresponding Author

Akira Mitsuhashi

Department of Reproductive Medicine

Graduate School of Medicine, Chiba University

Inohana 1-8-1, Chuo-ku, Chiba 260-8670, Japan

Tel: +81-43-226-2121

Fax: +81-43-226-2122

E-mail: a-mitsu@dokkyomed.ac.jp

Supplementary Information

Supplementary Table S1: Summary of gene-specific primer sequences for PCR

Supplementary Figure S1: The result of LDH releasing assay in RXRα or RXRβ knockdown cells

Supplementary Figure S2: Full-length western blot image for Fig. 2

Supplementary Figure S3: Full-length western blot image for Fig. 3

Supplementary Figure S4: Full-length western blot and agarose gel electrophoresis for Fig. 4.

Supplementary Table S1

Target	Forward Primer (5'-3')	Reverse Primer (5'-3')
RXRA	TTGCCAAGCAGCCGACAAACAG	AAGGAGGCGATGAGCAGCTCAT
RXRB	GCTGTGGAACAGAAGAGTGACC	CCCACTCAACAAGCGTGAATAGC
RXRG	GACTGCCTCATTGACAAGCGTC	GACCACTGGTAGCACATTCTGC
CDKN1A	AGGTGGACCTGGAGACTCTCAG	TCCTCTTGGAGAAGATCAGCCG
XBP1	CCTGGTTGCTGAAGAGGAGG	CCATGGGGAGATGTTCTGGAG
GAPDH	GCTCTCTGCTCCTCCTGTTC	ACGACCAAATCCGTTGACTC

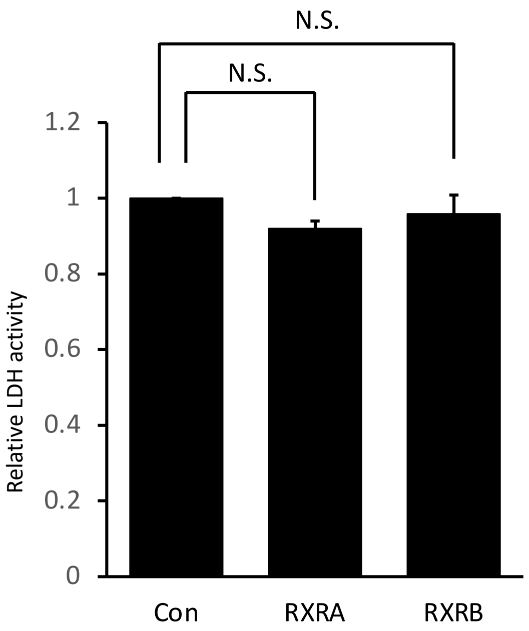

Figure S 1. RXRα and RXRβ were not related to bexarotene-induced pyroptosis in an ovarian cancer cell line.

After bexarotene treatment, extracellular LDH was measured and compared between the control group and RXRα or RXRβ siRNA-transfected group. Significant differences in LDH activities were not found between each siRNA-transfected group and the control group. Bar graphs represent the mean ± standard deviation of at least three independent experiments. RXR, Retinoid X receptor; NS, not significant.

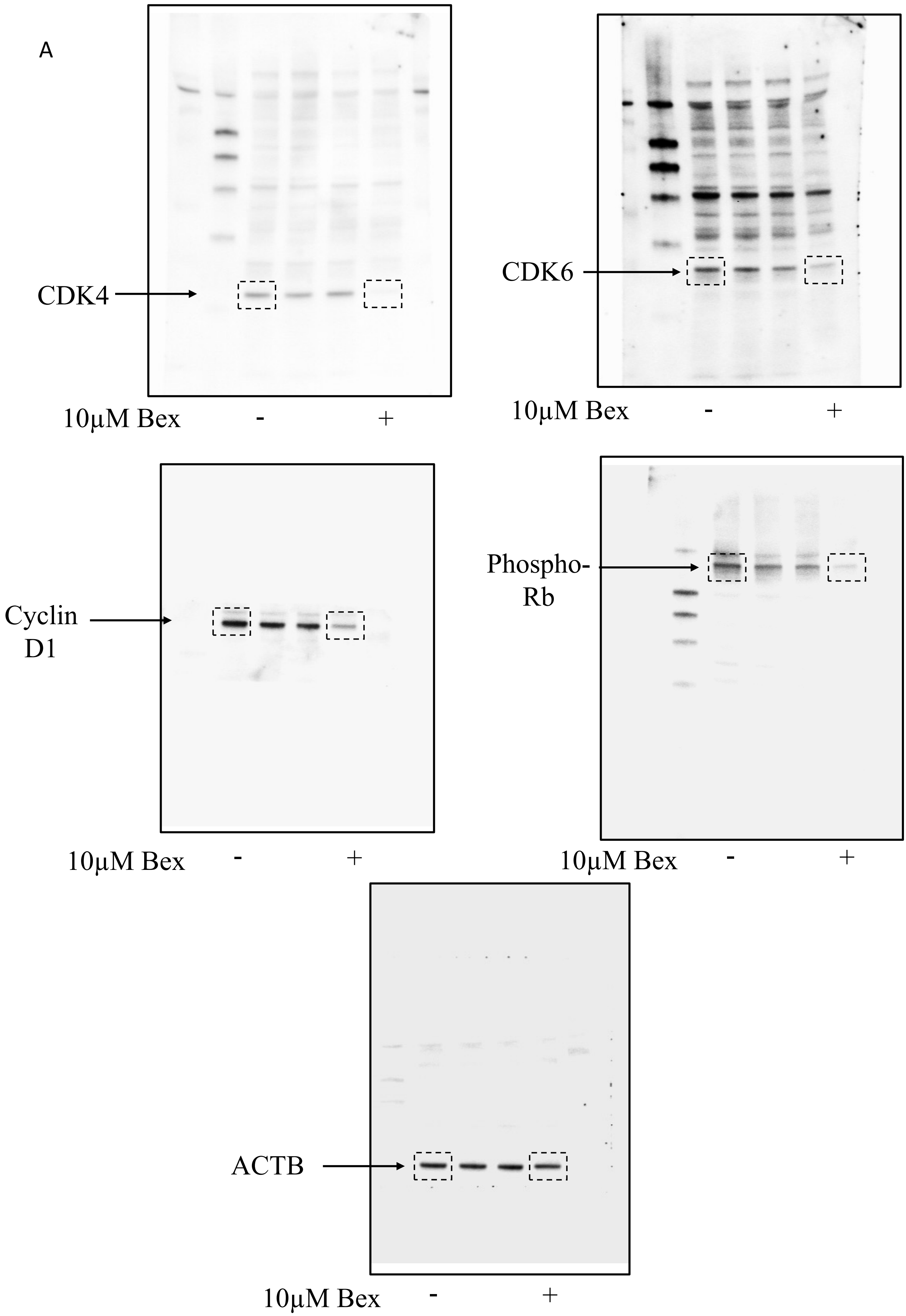

Figure S 2. Full-length Western blot image for Fig2. (A) full-lemngth image for figure 2c for ES2 cell (B) full-lemngth image for figure 2c for NIH: OVACAR3

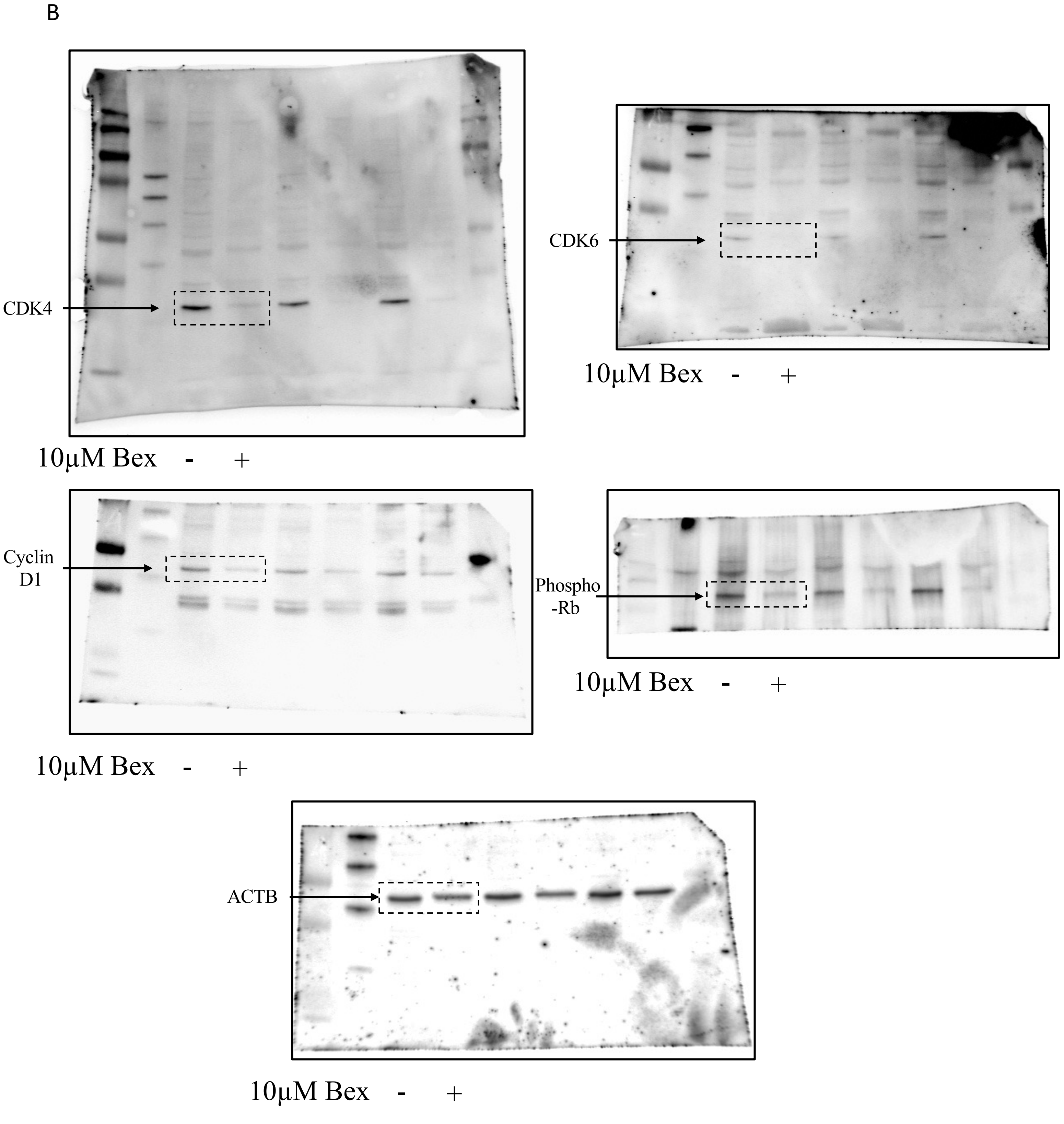

Figure S 2. Full-length Western blot image for Fig2. (A) full-lemngth image for figure 2c for ES2 cell (B) full-lemngth image for figure 2c for NIH: OVACAR3

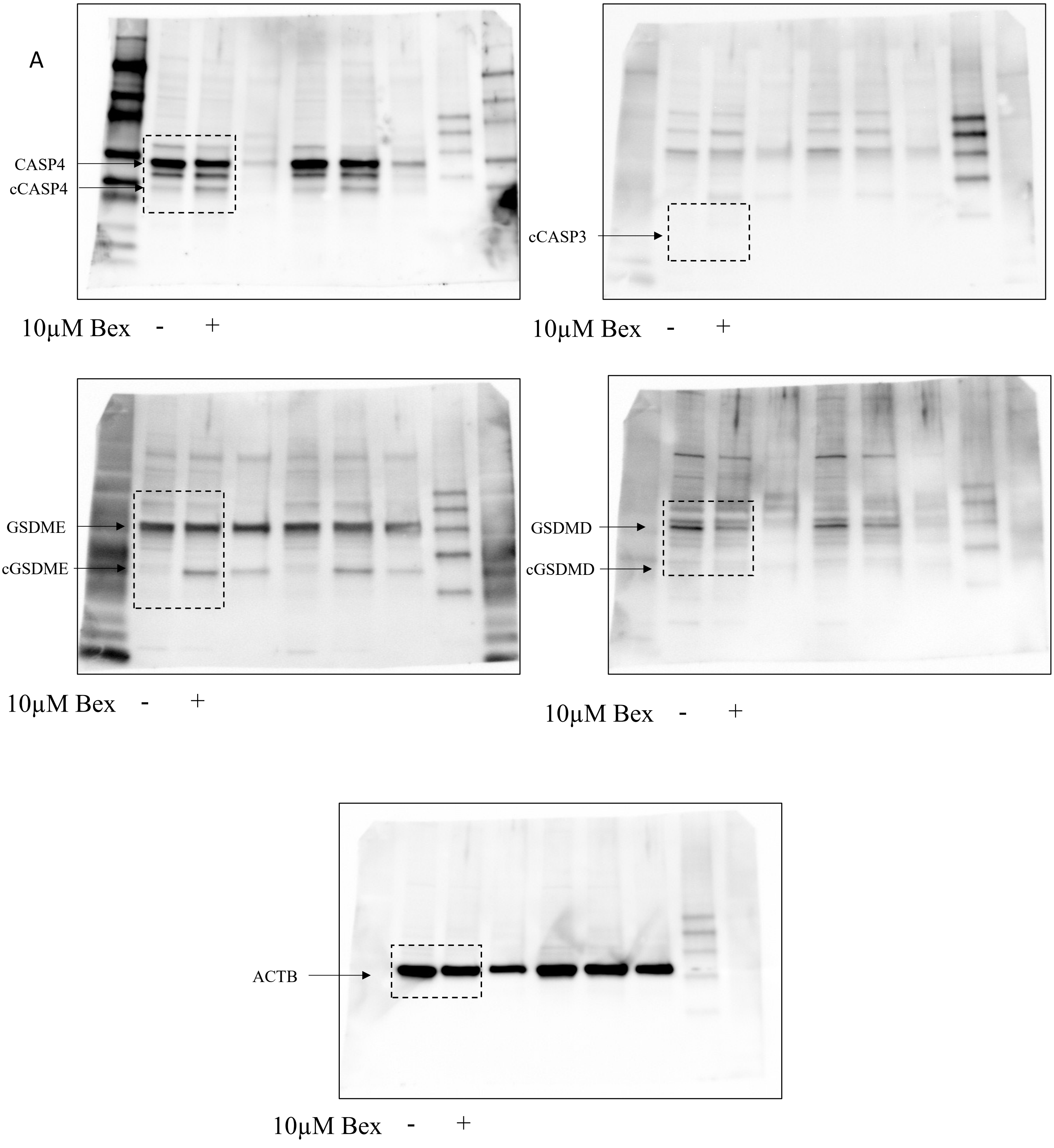

Figure S 3. Full-length Western blot image for Fig.3

B

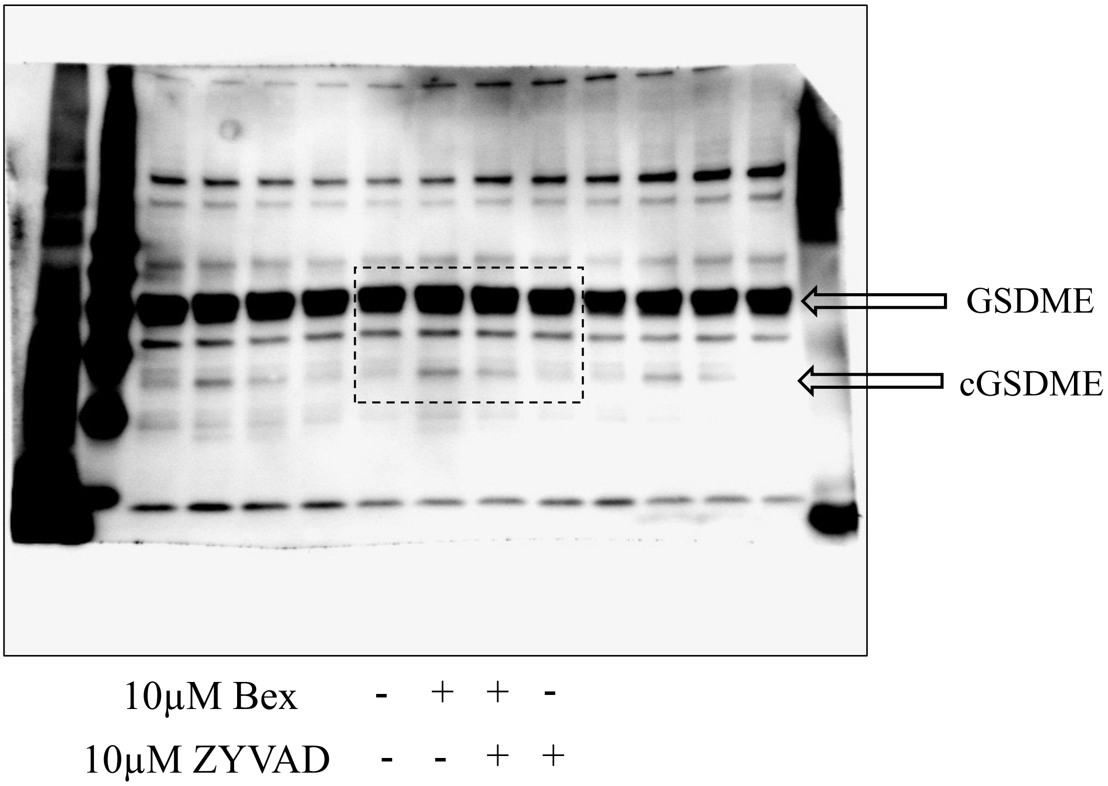

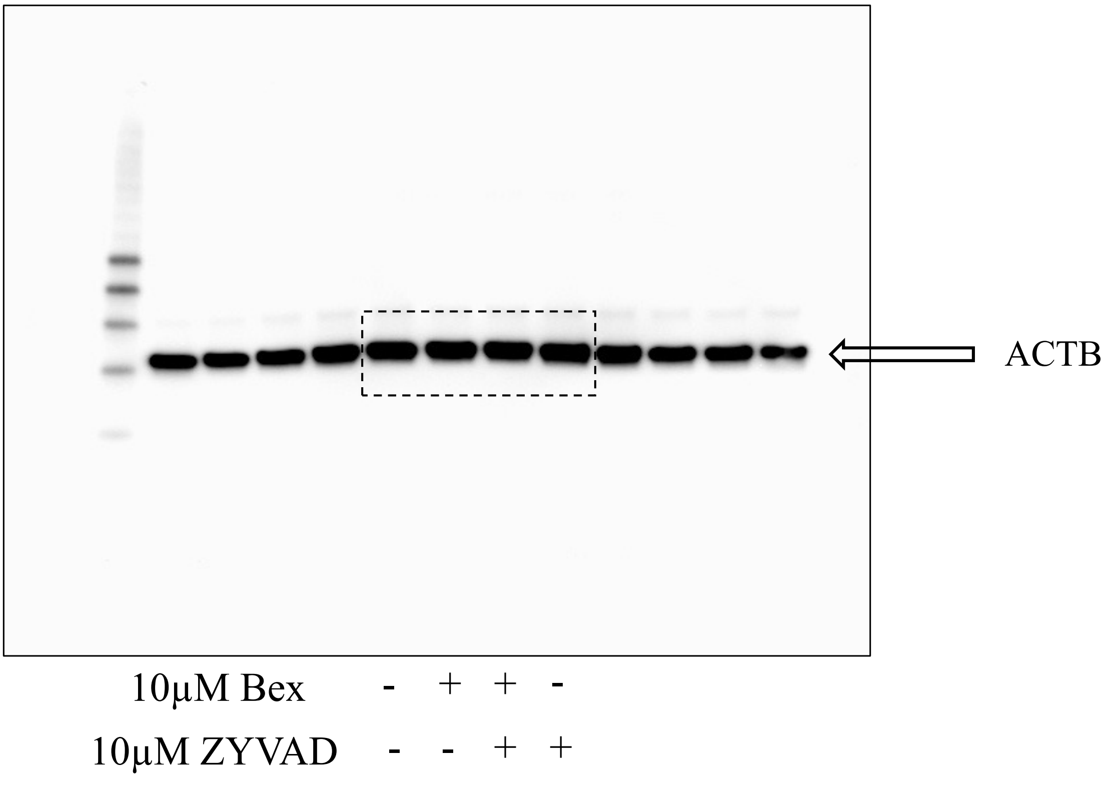

Figure S 3. Full-length Western blot image for Fig.3

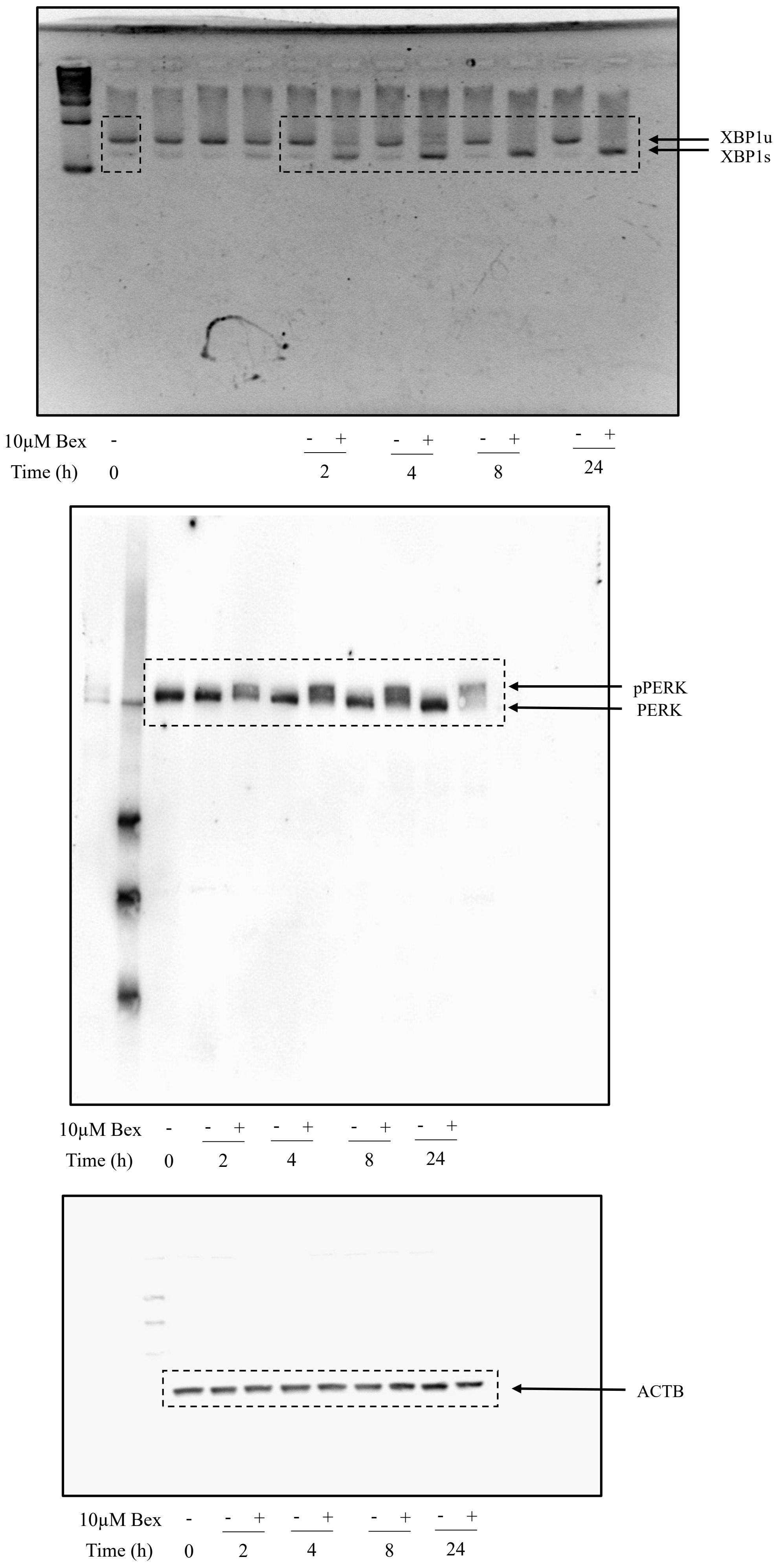

Figure S 4. Full-length Western blot and agarose gel electrophoresis.
